# Supplementary material for: Cytostatic Activity of Sanguinarine and a Cyanide Derivative in Human Erythroleukemia Cells Is Mediated by Suppression of c-MET/MAPK Signaling
Source: Int J Mol Sci. 2023 Apr 30;24(9):8113. doi: 10.3390/ijms24098113 (PMC10179035; doi:10.3390/ijms24098113)
Supplement: Supplementary file 1 [file ijms-24-08113-s001.zip › ijms-2341824-supplementary.pdf]

Supporting information

The cytostatic activity of sanguinarine and a cyanide derivative in human erythroleukemia cells is mediated by suppression of c-MET/MAPK signaling

Xinglian Xu,[a, b 1], Lulu Deng [a, b 1], Yaling Tang [b, c], Jiang Li [a, b], Ting Zhong [a, b], Xiaojiang Hao [a, b, c], Yanhua Fan [a, b **], and Shuzhen Mu [a, b *].

[a]*State Key Laboratory of Functions and Applications of Medicinal Plants, Guizhou Medical University, 3491 Beijin Road, Guiyang, Guizhou, 550014, People's Republic of China;*

[b]*The Key Laboratory of Chemistry for Natural Products of Guizhou Province and Chinese Academy of Sciences, 3491 Beijin Road, Guiyang, Guizhou, 550014, People's Republic of China;*

[c] *Kunming Institute of Botany, Chinese Academy of Sciences (CAS), Kunming 650201, China;*

Content

1. The chemical structure of sanguinarine and its derivatives **3**

2. The IC_{50} of of sanguinarine and its derivatives in HEL cells **4**

3. The pass word of the NCBI database for RNA-seq data **5**

4. The sequence of c-MET... **6**

5 Overexpression of c-MET in HEL cells **8**

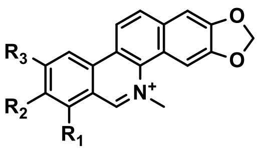

1. R_1+R_2=-CH_2-O-CH_2-, R_3=H, **Sanguinarine**
2. R_1=H,R_2=R_3=OMe, **Nitidine chloride**
3. R_1=R_2=OMe, R_3=H, **Chelerythrine**

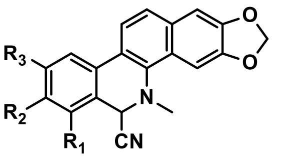

1a. R_1+R_2=-CH_2-O-CH_2-, R_3=H;
2a. R_1=H,R_2=R_3=OMe;
3a. R_1=R_2=OMe, R_3=H;

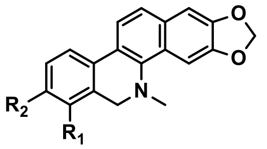

1b . R_1+R_2=-CH_2-O-CH_2-;
3b. R_1=R_2=OMe;

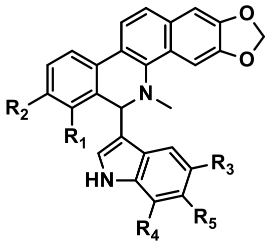

1c. R_1+R_2=-CH_2-O-CH_2-, R_3=-OH, R_4=R_5=-H;
3c. R_1=R_2=OMe, R_3=-OH, R_4=R_5=-H;
1d. R_1+R_2=-CH_2-O-CH_2-, R_3=-Cl,cR_4=R_5=-H;
3d. R_1=R_2=OMe, R_3=-Cl, R_4=R_5=-H;
3r. R_1=R_2=OMe, R_3=R_4=R_5=-H;
3s. R_1=R_2=OMe R_3=-NH_2, R_4=R_5=-H;
3t. R_1=R_2=OMe, R_3=R_5=-H, R_4=-Me;
3u. R_1=R_2=OMe, R_3=-F, R_4=R_5=-H;
3v. R_1=R_2=OMe, R_3=R_4=-H, R_5=-OMe;

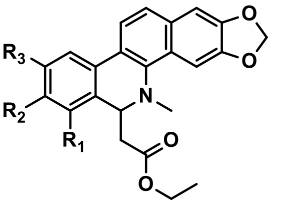

1e. R_1+R_2=-CH_2-O-CH_2-, R_3=H;
2e. R_1=H, R_2=R_3=OMe;
3e. R_1=R_2=OMe, R_3=H;

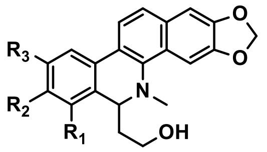

1f. R_1+R_2=-CH_2-O-CH_2-, R_3=H;
2f. R_1=H, R_2=R_3=OMe;
3f. R_1=R_2=OMe, R_3=H;

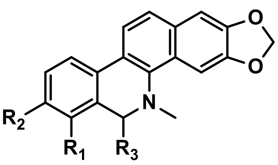

1g. R_1+R_2=-CH_2-O-CH_2-, R_3=
3g. R_1=R_2=OMe, R_3=
1h. R_1+R_2=-CH_2-O-CH_2-, R_3=
3h. R_1=R_2=OMe, R_3=

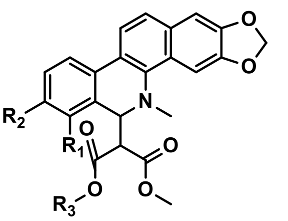

1i-1l. R_1+R_2=-CH_2-O-CH_2-, R_3=-Me,-Et, -Pro, -n-Bu
3i-3l. R_1=R_2=OMe, R_3=-Me, -Et,-Pro, -n-Bu

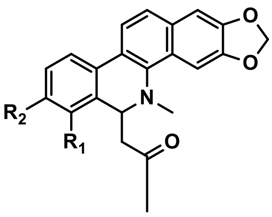

3m. R_1=R_2=OMe

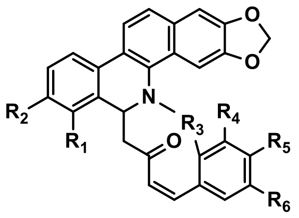

3n. R_1=R_2=OMe, R_4=-OMe, R_3=R_5=R_6=-H;
3o. R_1=R_2=OMe, R_4=-Me, R_3=R_5=R_6=-H;
3p. R_1=R_2=OMe, R_4=-Br, R_3=R_5=R_6=-H;
3q. R_1=R_2=OMe, R_3=-Br, R_5=R_6=-OMe, R_4=-H;

Figure S1. The chemical structure of sanguinarine and its derivatives

Table S1. Antiproliferative activities (IC_{50} values in μM) of sanguinarine and derivatives against HEL Cell Line.

**Compd.**	**IC_{50}(μM)**	**Compd.**	**IC_{50}(μM)**	**Compd.**	**IC_{50}(μM)**
**1**	0.96 ± 0.15	**2a**	5.59 ± 0.43	**3k**	14.15 ± 1.0
**1a**	1.05 ± 0.26	**2b**	17.41 ± 5.2	**3l**	13.18 ± 0.3
**1b**	>20	**2c**	>20	**3m**	>20
**1c**	19.46 ± 7.4	**3**	3.60 ± 0.34	**3n**	>20
**1d**	>20	**3a**	11.15 ± 1.5	**3o**	>20
**1e**	>20	**3b**	>20	**3p**	>20
**1f**	>20	**3c**	>20	**3q**	>20
**1g**	>20	**3d**	18.32 ± 3.0	**3r**	>20
**1h**	>20	**3e**	>20	**3s**	>20
**1i**	7.01 ± 1.09	**3f**	>20	**3t**	>20
**1j**	2.31 ± 0.42	**3g**	>20	**3u**	>20
**1k**	2.74 ± 0.30	**3h**	>20	**3v**	>20
**1l**	2.69 ± 1.06	**3i**	13.43 ± 0.3	Doxorubicin hydrochloride	0.27±0.02
**2**	14.15 ± 2.1	**3j**	14.52 ± 0.8		

Table S2. The pass word of the NCBI database for RNA-seq data.

Account	Pass word
2500297887@qq.com	xuxinglian999

Table S3. The sequence of c-MET.

Gene	Sequence	Species
c-MET	TTTGTTAGACGAAGCTTGGGCTGCAGGTCGACTCTAG<br>AGGATCCCCGGGTACCGGTCGCCACCATGGCGGAGC<br>CGAGCGGCTCGCCCGTGCACGTCCAGCTTCCCCAGC<br>AGGCGGCCCCGGTGACAGCGGCGGCGGCGGCC<br>CCGGCGGCCGCGACAGCAGCGCCGGCCCCGGCAGCT<br>CCCGCGGCCCCGGCCCCGGCCCCGGCCCCGGCGGCA<br>CAGGCTGTCGGCTGGCCCATCTGCAGGGACGCGTAC<br>GAGCTGCAGGAGGTTATCGGCAGTGGAGCTACTGCT<br>GTGGTTCAGGCAGCCCTATGCAAACCCAGGCAAGAA<br>CGTGTAGCAATAAAACGGATCAACTTGGAAAAATGCC<br>AGACCAGTATGGATGAACTATTAAAAGAAATTCAAGC<br>CATGAGTCAGTGCAGCCATCCCAACGTAGTGACCTAT<br>TACACCTCTTTTGTGGTCAAAGATGAACTTTGGCTGG<br>TCATGAAATTACTAAGTGGAGGTTCAATGTTGGATATC<br>ATAAAATACATTGTCAACCGAGGAGAACACAAGAAT<br>GGAGTTCTGGAAGAGGCAATAATAGCAACAATTCTTA<br>AAGAGGTTTTGGAAGGCTTAGACTATCTACACAGAA<br>ACGGTCAGATTCACAGGGATTTGAAAGCTGGTAATAT<br>TCTTCTGGGTGAGGATGGTTCAGTACAAATAGCAGAT<br>TTTGGGGTAAGTGCGTTCCTAGCAACAGGGGGTGATG<br>TTACCCGAAATAAAGTAAGAAAAACATTCGTTGGCAC<br>CCCATGTTGGATGGCTCCTGAAGTCATGGAACAGGTG<br>AGAGGCTATGACTTCAAGGCTGACATGTGGAGTTTTG<br>GAATAACTGCCATTGAATTAGCAACAGGAGCAGCGCC<br>TTATCACAAATATCCTCCCATGAAAGTGTTAATGTTGA<br>CTTTGCAAAATGATCCACCCACTTTGGAAACAGGGGT<br>AGAGGATAAAGAAATGATGAAAAAGTACGGCAAGTC<br>CTTTAGAAAATTACTTTCACTGTGTCTTCAGAAAGAT<br>CCTTCCAAAAGGCCCACAGCAGCAGAACTTTTAAAA<br>TGCAAATTCTTCCAGAAAGCCAAGAACAGAGAGTAC<br>CTGATTGAGAAGCTGCTTACAAGAACACCAGACATA<br>GCCCAAAGAGCCAAAAAGGTAAGAAGAGTTCCTGGG<br>TCAAGTGGTCACCTTCATAAAACCGAAGACGGGGAC<br>TGGGAGTGGAGTGACGACGAGATGGATGAGAAGAGC<br>GAAGAAGGGAAAGCAGCTTTTTCTCAGGAAAAGTCA<br>CGAAGAGTAAAAGAAGAAAATCCAGAGATTGCAGTG<br>AGTGCCAGCACCATCCCCGAACAAATACAGTCCCTCT<br>CTGTGCACGACTCTCAGGGCCCACCCAATGCTAATGA<br>AGACTACAGAGAAGCTTCTTCTTGTGCCGTGAACCTC<br>GTTTTGAGATTAAGAAACTCCAGAAAGGAACTTAATG<br>ACATACGATTTGAGTTTACTCCAGGAAGAGATACAGC	Human

AGATGGTGTATCTCAGGAGCTCTTCTCTGCTGGCTTG
GTGGATGGTCACGATGTAGTTATAGTGGCTGCTAATTT
ACAGAAGATTGTAGATGATCCCAAAGCTTTAAAAACA
TTGACATTTAAGTTGGCTTCTGGCTGTGATGGGTCGG
AGATTCCTGATGAAGTGAAGCTGATTGGGTTTGCTCA
GTTGAGTGTCAGCCCGGTCGCCACCATGGTGAGCAA
G G G

A B C

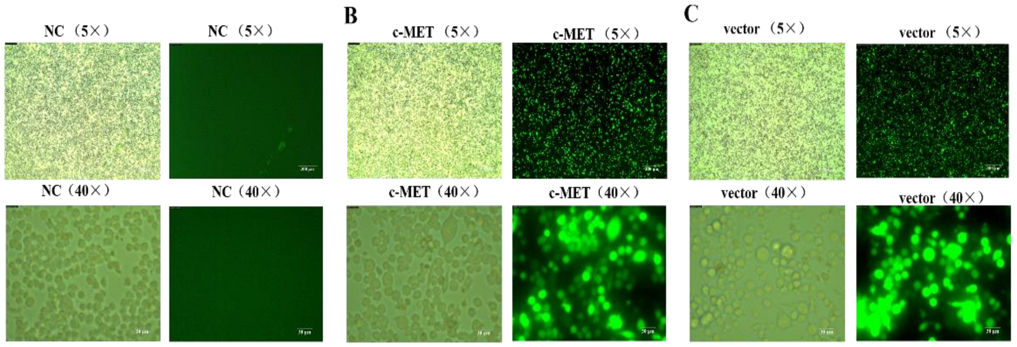

D

Type of virus	Infection rate of lentivirus (%)
negative control (NC)	0
c-MET (LV-MET (73111-1))	81.78±2.34
vector (CON 335)	82.82±3.12

E

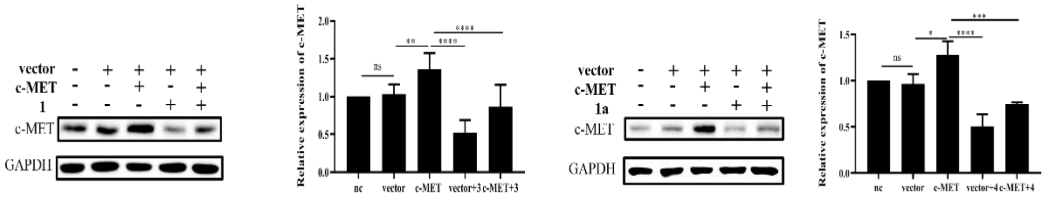

Figure S2. Overexpression of c-MET in HEL cells. (A) Fluorescence expression of normal HEL cells. Scale bar: 300 μm (5×), 30 μm (40×). (n = 3). (B) Fluorescence expression of HEL cells infected by LV-MET (73111-1) virus uder different microscopic magnifications. Scale bar: 300 μm (5×), 30 μm (40×). (n = 3). (C) Fluorescence expression of HEL cells infected by empty vector virus (CON 335) under different microscopic magnifications. Scale bar: 300 μm (5×), 30 μm (40×). (n = 3). (D) Fluorescence expression rate of HEL cells. (E) The protein level of c-MET after overexpression c-MET assay and compounds treated.
